# MUC17 and the intestinal glycocalyx: structure, regulation, and barrier function in health and disease

**DOI:** 10.1042/BSR20250123

**Published:** 2026-10-01

**Authors:** Fleur Claire van de Koolwijk, Thaher Pelaseyed

**Affiliations:** Department of Medical Biochemistry and Cell Biology, Institute of Biomedicine, University of Gothenburg, Box 440, 405 30 Gothenburg, Sweden

**Keywords:** barrier, glycocalyx, MUC17, Transmembrane mucin

## Abstract

The intestinal epithelial surface is protected by a multilayered barrier system that includes secreted mucus and a membrane-bound glycocalyx. While gel-forming mucins have been extensively studied, the transmembrane mucins that form the enterocyte glycocalyx remain less well understood. MUC17 has emerged as a dominant and functionally important transmembrane mucin of the small intestinal brush border, where its large, extensively *O*-glycosylated extracellular domain forms a dense, hydrated barrier between luminal microbes and the epithelial membrane. This review summarizes current knowledge of MUC17 biology, from genomic organization and protein architecture to glycosylation, trafficking, developmental regulation, and barrier function. Particular emphasis is placed on how MUC17 contributes to size-selective exclusion, bacterial segregation, and host-microbe homeostasis. This review further discusses the developmental establishment of the MUC17-rich enterocyte glycocalyx during the suckling–weaning transition and covers the mechanisms that regulate MUC17 localization and dynamics in epithelial cells, including apical targeting, membrane retention, recycling, turnover, and responses to inflammatory cues. Finally, the review examines the consequences of altered MUC17 expression or function, with particular emphasis on glycocalyx barrier defects, host-microbe interactions, bacterial access to the epithelium, and the relevance of MUC17 dysfunction to Crohn’s disease and experimental models of intestinal infection. Together, the review positions MUC17 as a central structural and functional component of the intestinal glycocalyx and an underexplored therapeutic axis for restoring epithelial barrier integrity in intestinal disease.

## Introduction: the glycocalyx as a critical epithelial barrier

The intestinal barrier is a multilayered defense system that protects the host from the luminal environment while maintaining selective permeability for nutrient uptake. While much attention has focused on the intestinal mucus layer formed by the secreted gel-forming mucin MUC2 [1], an equally important but less explored component resides directly at the epithelial surface: the glycocalyx formed by transmembrane mucins. These membrane-anchored glycoproteins extend from the apical membrane of enterocytes, creating a dense, hydrated interface that constitutes the final barrier between host cells and luminal microbes. These two mucin-based systems therefore act at different distances from the epithelial membrane but together shape the host-luminal interface.

In the small intestine, where the mucus layer is relatively permeable, transmembrane mucins play a particularly critical role in maintaining epithelial protection [2]. By forming a cell-attached glycocalyx, they regulate molecular diffusion, prevent direct microbial contact, and define the physicochemical properties of the epithelial surface. Among the transmembrane mucins, MUC17 has emerged as a dominant component of the small intestinal glycocalyx, characterized by its extensive *O*-glycosylation and ability to project far into the lumen. Despite its central position at the host-microbe interface, the biology of MUC17 and the glycocalyx to which it contributes remains incompletely understood.

Recent advances have begun to reveal that transmembrane mucins are not passive structural elements but dynamic and regulated components of an innate epithelial defense system. This review focuses on MUC17 as a model transmembrane mucin, integrating insights from gene to protein to physiology to better understand how the glycocalyx contributes to intestinal homeostasis. Here, we use the term enterocyte glycocalyx to describe the higher-order, hydrated cell-surface layer formed by transmembrane mucins and other membrane-associated glycoconjugates. MUC17 is a major molecular constituent of this structure in the small intestine. Accordingly, we refer to a MUC17-rich glycocalyx when describing its composition and to a MUC17-dependent glycocalyx barrier when referring to functions that depend on MUC17.

## The mucin family and classification of transmembrane mucins

Mucins constitute a large and diverse family of high-molecular-weight glycoproteins that are central to the protection of mucosal surfaces [3]. Despite considerable heterogeneity in size, domain composition, and tissue distribution, all mucins share a defining structural feature: an extended mucin domain rich in proline, threonine, and serine (PTS) residues [4]. The serine and threonice residues serve as substrates for *O*-linked glycosylation, generating a highly hydrated, protease-resistant mucin domain that forms the biochemical foundation of the mucosal barrier. Based on their overall architecture and mode of deployment at epithelial surfaces, mucins are broadly divided into two major classes: secreted, gel-forming mucins and membrane-bound transmembrane mucins.

Gel-forming mucins, including MUC2, MUC5AC, MUC5B, and MUC6, are predominantly polymeric proteins secreted by goblet and mucus-secreting cells that assemble into large viscoelastic networks [4]. These polymers form the mucus gel that overlays epithelial surfaces and acts as the first line of defense by trapping microbes and limiting their access to the epithelium [5]. In contrast, transmembrane mucins are monomeric, single-pass type I transmembrane proteins that remain anchored to the plasma membrane of cells [6]. Rather than forming a secreted gel, these mucins extend directly from the cell surface into the lumen, creating a dense glycosylated layer that constitutes a physical and biochemical interface between host cells and the external environment.

Within transmembrane mucins, two subclasses can be defined based on the presence of specific extracellular domains. Of particular relevance to intestinal biology is the group of SEA domain-containing intestinal transmembrane mucins, including MUC1, MUC3A, MUC3B, MUC12, MUC16 and MUC17. These mucins share a conserved domain organization consisting of a large extracellular PTS-rich mucin domain, a juxtamembrane sea urchin sperm protein, enterokinase, and agrin (SEA) domain, a single transmembrane domain, and a cytoplasmic tail [2].

The extensive O-glycosylation of the PTS-rich domain is the key determinant of mucin function. The dense array of glycans transforms the protein backbone into a rigid, bottle brush-like structure that projects far into the lumen, often reaching lengths of up to a micrometer. This creates a highly hydrated and sterically hindered barrier that limits diffusion of macromolecules, prevents direct microbial contact with the epithelial surface, and provides binding sites for microbial adhesins and host factors.

In the intestinal tract, transmembrane mucins are prominently expressed by enterocytes, where they form the glycocalyx coating the apical brush border [7]. Within this group, MUC17 has emerged as a dominant and functionally critical constituent of the small intestinal glycocalyx, contributing to the establishment of a dense, cell-attached barrier. Understanding MUC17 therefore requires its placement within this broader family of transmembrane mucins, whose shared structural principles underpin a specialized and dynamic defense system at mucosal surfaces.

## Genomic organization and evolution of the MUC17 locus

The *MUC17* gene sits within a conserved mucin gene cluster on human chromosome 7q22, together with *MUC3A*, *MUC3B*, and *MUC12* (Figure 1A). This locus encodes a group of closely related transmembrane mucins that share common structural features, including a large PTS-rich extracellular domain encoded by a single exon and a juxtamembrane SEA domain. The physical clustering of these genes is thought to reflect their origin through gene duplication events and their subsequent specialization for functions at mucosal surfaces [8–10]. Notably, this genomic arrangement appears to be conserved during mammalian evolution, with the *MUC3A-MUC3B–MUC12–MUC17* cluster typically positioned between *ACHE* upstream and *TRIM56* downstream (Figure 1B,C) [8]. Comparative genomic analyses indicate that this mucin cluster is evolutionarily conserved across mammals, although its organization and annotation have been challenging [8]. A notable example is the murine ortholog of human *MUC17*, historically referred to as *Muc3* due to sequence similarity and genomic proximity. This has led to the designation Muc3(17) to clarify its orthology to human MUC17. Such discrepancies highlight broader issues in mucin gene annotation, which stem from the highly repetitive nature of PTS-rich domains. These regions are difficult to sequence and assemble accurately, often resulting in incomplete gene models, collapsed repeats, or misassignment of orthologous relationships [11]. Recent advances in long-read sequencing have begun to resolve these complexities, revealing previously unrecognized genes (e.g. *MUC3B*) and refining our understanding of the locus [8,12].

**Figure 1:**
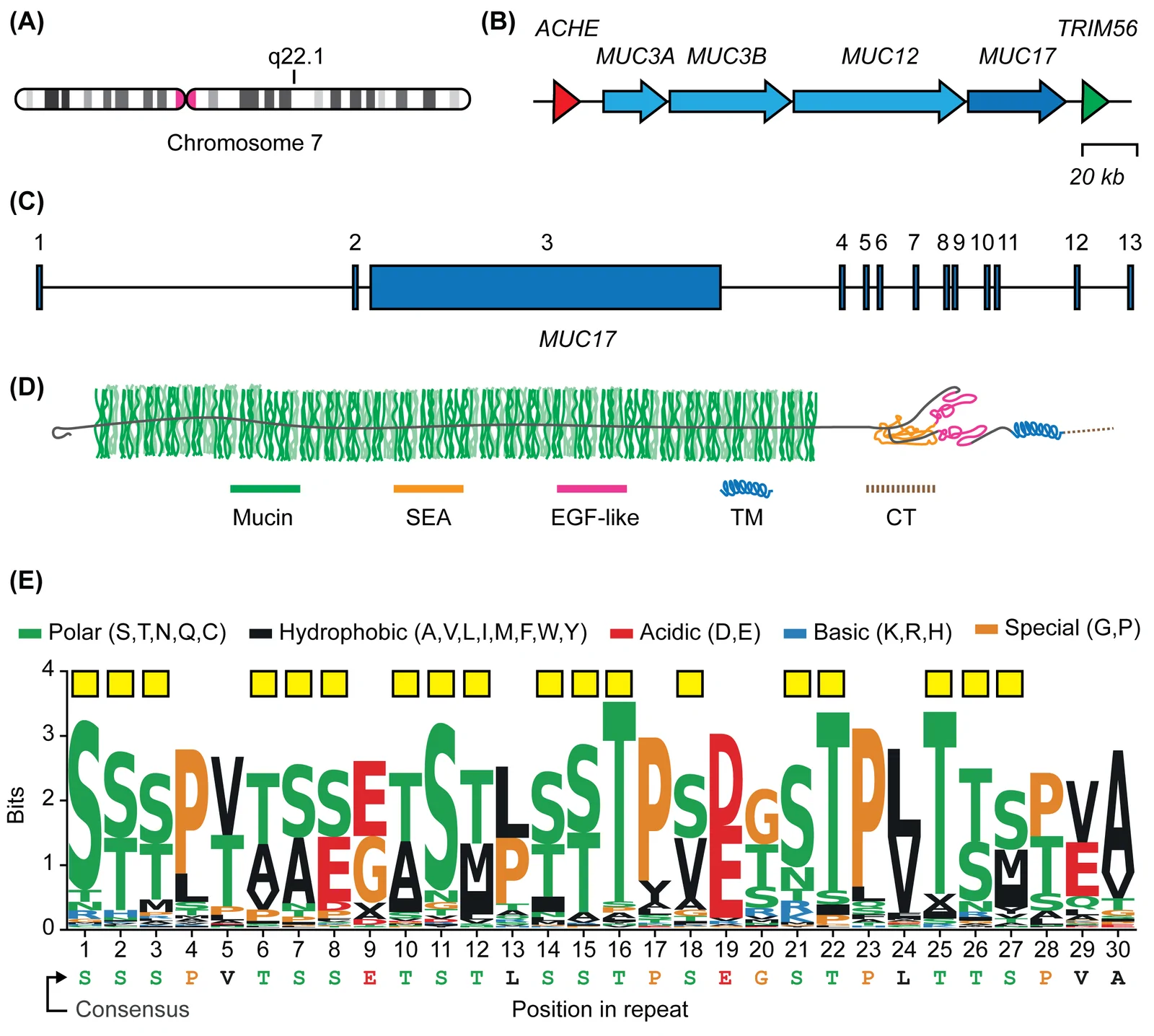
Genomic organization, protein architecture, and PTS repeat composition of MUC17 (**A**) Localization of the *MUC17* gene locus on human chromosome 7q22.1. (**B**) Organization of the intestinal membrane mucin gene cluster, showing ACHE, MUC3A, MUC3B, MUC12, MUC17, and TRIM56. Scale bar, 20 kb. (**C**) Exon structure of the *MUC17* gene. (**D**) Schematic domain organization of MUC17, including the mucin domain, SEA domain, EGF-like domains, transmembrane domain, and cytoplasmic tail. (**E**) Consensus sequence logo of the MUC17 PTS repeat sequence, showing amino acid enrichment across the repeat. Amino acids are colored according to physicochemical class. GalNAc residues (yellow squares) indicate putative *O*-glycosylation sites.

The evolutionary expansion of the *MUC3A-MUC3B–MUC12–MUC17* cluster likely reflects selective pressures imposed by the intestinal environment, where epithelial cells must continuously adapt to a dense and dynamic microbial ecosystem. Diversification of these genes may have enabled functional specialization, such as differences in expression patterns along the gastrointestinal tract, glycosylation profiles, or interactions with microbial and host factors. In this context, MUC17 appears to have evolved as a dominant component of the small intestinal glycocalyx, consistent with its high expression in enterocytes and its critical role in maintaining barrier integrity.

Genetic variation within the MUC17 locus may also have functional consequences for epithelial defense and disease susceptibility. While large-scale genetic studies of MUC17 within the population remain limited, alterations in mucin gene expression and structure have been associated with inflammatory bowel disease and other mucosal disorders. Given the importance of MUC17 in preventing bacterial contact with the epithelium, polymorphisms affecting its length and glycosylation could influence host–microbe interactions and predispose individuals to inflammation. Thus, the genomic organization of the MUC17 locus not only reflects its evolutionary history but also provides a framework for understanding how genetic variation may impact intestinal health and disease.

## MUC17 protein architecture: domain organization and biochemical properties

### PTS-rich sequences and the *O*-glycosylated mucin domain

Mucins all share one common feature: a heavily *O*-glycosylated mucin domain made up of tandem repeats (TRs) rich in the amino acids proline, serine, and threonine [6,13–15] (Figure 1D). Since the repetitive nature of TRs makes them prone to recombination and polymorphism, the domain is also referred to as a variable number tandem repeat (VNTR) domain. MUC17 has a PTS-rich domain composed of 4073 amino acids, making up a total of 60 TRs consisting of ∼12% prolines, 23% serines, and 26% threonines (Figure 1E). The numerous *O*-glycans force the protein backbone into a linear, bottle brush-like conformation, extending up to 1 μm into the lumen [16,17]. As the folded protein moves through the Golgi, the serines and threonines are extensively *O*-glycosylated [18,19], accounting for up to 80% of total mass of MUC17 (Figure 1E) [20]. Mucin-type *O*-glycosylation is initiated by polypeptide N-acetylgalactosaminyltransferases (ppGalNAcTs), which add N-acetylgalactosamine (GalNAc) to serine or threonine residues [21,22]. This initial GalNAc residue, known as the Tn antigen, can then be extended into several distinct core structures rather than following a single linear pathway [23]. Addition of galactose by core 1 β1,3-galactosyltransferase generates the core 1 structure, Galβ1–3GalNAcα-Ser/Thr, a reaction that requires the molecular chaperone Cosmc for proper enzyme folding and activity [21]. Core 1 can be further branched by addition of GlcNAc in β1–6 linkage to form core 2, Galβ1–3[GlcNAcβ1–6]GalNAcα-Ser/Thr. Alternatively, addition of GlcNAc in β1–3 linkage to GalNAc generates core 3, GlcNAcβ1–3GalNAcα-Ser/Thr, which is widely distributed in human intestinal mucins and represents a major scaffold for further elongation [24,25]. Core 3 can in turn be branched to form core 4, GlcNAcβ1–3[GlcNAcβ1–6]GalNAcα-Ser/Thr.

The pattern of *O*-glycans differs along the intestinal tract, reflecting, at least in part, differential expression of GalNAcTs and other glycosyltransferases, while microbiota- and diet-dependent signals can further shape mucin *O*-glycan patterns through effects on the epithelial glycosylation machinery [26–29]. Structural analyses of purified human intestinal mucins have shown that highly fucosylated glycans found in the ileum are mainly based on core 4 structures, whereas core 2-based sulfo-Lewis X-containing glycans are more characteristic of the distal colon. Core 5 structures, GalNAcα1–3GalNAcα-Ser/Thr, have also been detected in human intestinal mucins, although they appear less prominent than core 3- and core 4-derived structures.

After *O*-glycosylation in the Golgi, the fully glycosylated mucin is trafficked to the apical membrane, a process that in turn is regulated by *O*-glycosylation [30]. The dense layer of glycans is crucial for protecting the mucin backbone against bacterial and enzymatic degradation [31,32]. At the same time, *O*-glycans are known to be utilized by microbial communities in the intestines by direct foraging and cross-feeding through sugars liberated by other microbes [33–40]. The interaction between bacteria and mucin *O*-glycans has been suggested to play a role in the selectionof certain commensals in different regions of the gut. Altered glycosylation has also been shown to be related to diseases of the intestine, such as ulcerative colitis and Crohn’s disease [41–43]. Thus, glycans present on the mucin domain are not only structural but also determine mucin extension, hydration, protease resistance, microbial binding, and regional host-microbe interactions.

### The SEA domain

All transmembrane mucins, except MUC4, MUC21, and MUC22, contain a SEA domain positioned extracellularly, immediately proximal to the transmembrane domain, linking the large *O*-glycosylated α-chain to the membrane-anchored β-chain (Figure 1D). MUC1, MUC3A, MUC3B, MUC12, MUC16, and MUC17 share the highly conserved SEA domain cleavage motif G↓S[V/I]VV [44,45]. Even though there has been limited published work on the MUC17 SEA domain, more has been published on the corresponding domain in MUC1. During protein folding in the endoplasmic reticulum, conformational stress on the Gly1097-Ser1098 residues combined with catalytic action from the Ser1098 hydroxyl leads to a strain-dependent autoproteolytic cleavage of the SEA domain [46,47]. Cleavage of the protein results in an extracellular α-chain and plasma membrane-attached β-chain that remain noncovalently associated by parallel β-sheets [46,48]. The SEA domain contains several N-glycosylation sites, which likely play a role in its folding [49]. The SEA domain can unfold in response to mechanical forces [50], suggesting that dissociation of the α- and β-chain participates in the protection of the plasma membrane against mechanical stress and shear forces exerted on the cell. Furthermore, the SEA domain has also been suggested to be involved in shedding of the mucin extracellular domain [51]. In mice, excessive shedding of Muc17 into the intestinal lumen was observed in a cystic fibrosis (CF) model [52,53]. In addition, other SEA domain-containing transmembrane mucins are known to be excessively shed into serum of patients with breast cancer, into culture supernatant by CFTR-deficient pancreatic cell lines mimicking active CF, and into the intestinal lumen of patients with Crohn’s disease [54–56]. Thus, these findings point towards a key role for the SEA domain in excessive shedding observed in disease, while potentially acting as a cell-protective device against mechanical stress under homeostatic conditions.

### The EGF-like domains

MUC17 carries two EGF-like domains, flanking the SEA domain (Figure 1D) [57,58]. Each EGF-like domain consists of six cysteine residues of which the first is less conserved, while the latter five are highly conserved between different transmembrane mucins [59]. These domains are thus also referred to as cysteine-rich domain 1 and 2. Although the exact function of the EGF-like domains is not fully defined, such domains are typically involved in mediating protein-protein interactions [60,61]. Early studies suggested a role as ligands for the EGF receptor (EGFR); however, evidence indicates that MUC17 does not directly activate EGFR but rather engages downstream ERK signaling pathways [62]. Interestingly, the β chain of MUC17, which retains the EGF-like domain following autoproteolytic cleavage of the SEA domain, has been shown to interact with ERBB2 (also known as human epidermal growth factor receptor 2, HER2) [63]. HER2 is a receptor tyrosine kinase of the same family as EGFR and a known proto-oncogene [63–65]. Although prior studies have not directly established that this interaction is mediated by the EGF-like domain of MUC17, both MUC4 and MUC13 are known to bind HER2 via their EGF-like domains [66–68]. It is important to note that many of these interactions have been described in cancer cell models, where epithelial polarity is frequently disrupted. Under homeostatic conditions, intestinal transmembrane mucins are largely confined to the apical surface, whereas receptor tyrosine kinases such as HER2 may be less accessible within the polarized epithelial architecture. In cancer, loss of polarity and redistribution of transmembrane mucins over the cell surface may bring mucin EGF-like domains into closer proximity with HER2 and other ErbB receptors, thereby facilitating interactions that may not occur, or may be spatially restricted, in healthy polarized epithelia. Notably, ERK is downstream of the HER2 signaling cascade [69], thus strengthening the notion that MUC17 EGF-like domains are responsible for its previously mentioned association with HER2. In addition, MUC17 EGF-like domains have been shown to promote cell migration and inhibit apoptosis [70]. Accordingly, these findings point towards a complex role for MUC17 EGF-like domains in promoting cell proliferation and healing of epithelial injury, while also raising the possibility of oncogenic signaling in pathological contexts. However, a concrete role for MUC17 EGF-like domains remains understudied.

## The cytoplasmic tail

The cytoplasmic tail of MUC17 is short compared with its large extracellular mucin domain, but it plays an important role in regulating the localization and dynamics of the protein at the apical membrane. A key feature of this domain is a conserved class I PDZ-binding motif, which mediates interaction with the scaffolding protein PDZK1 [71,72]. This interaction is highly specific among the chromosome 7 transmembrane mucins. Although MUC3A/B, MUC12, and MUC17 all contain class I PDZ-binding motifs, PDZK1 interacts specifically with the cytoplasmic tail of MUC17, whereas comparable binding to the cytoplasmic tails of MUC3 or MUC12 was not detected in pull-down experiments. The interaction depends on the extreme C-terminal residues of MUC17 and involves multiple PDZ domains within PDZK1. Functionally, PDZK1 stabilizes MUC17 at the enterocyte brush border, as loss of Pdzk1 in mouse jejunum delocalizes Muc17 from the apical membrane to intracellular vesicular structures. The cytoplasmic tail of MUC17 also contains serine residues that can be phosphorylated, suggesting that post-translational modification may further regulate MUC17 interactions, apical retention, endocytosis, or recycling [73]. Thus, although the extracellular domain provides the structural basis for glycocalyx barrier function, the cytoplasmic tail is essential for positioning and dynamically regulating MUC17 at the epithelial surface.

## The biophysical properties of the MUC17 mucin domain

The defining functional feature of MUC17 is its extensively *O*-glycosylated mucin domain. This dense glycan decoration imparts unique and highly organized biophysical properties. At the molecular level, steric hindrance and electrostatic repulsion between closely spaced and negatively charged glycan moieties force the mucin backbone into an extended, bottle brush-like conformation. At the supramolecular level, these individual transmembrane mucins assemble into micrometer-long filaments that project from microvillar tips into the intestinal lumen, forming a continuous glycocalyx layer approximately 1 μm thick [17]. These filaments organize into a three-dimensional columnar network with an average pore size of ∼30 nm, creating a highly ordered meshwork that functions as a size-selective diffusion barrier.

The architecture of the glycocalyx is not merely structural but directly governs microbial interactions. The dense array of *O*-glycans displayed along the mucin domain constitutes a complex “glycocode” that is recognized by microbial adhesins and enzymes [74]. Bacterial binding to MUC17 is therefore mediated by the recognition and specificity of these adhesins for host glycan epitopes, often in patches of multiple adjacent carbohydrate chains [75,76]. Importantly, these bacteria-mucin interactions are inherently dual in nature: while glycans can serve as binding sites that capture bacteria, the extended and hydrated glycocalyx simultaneously increases the physical distance between microbes and the host cell membrane, thereby limiting productive adhesion and further detrimental challenge to the host.

Functionally, MUC17 can be envisioned as a spatially resolved diffusion barrier and decoy system. Here, decoy refers to the ability of the extended, glycosylated extracellular domain of MUC17 to present bacterial binding sites away from the epithelial plasma membrane. Thus, bacteria that recognize mucin *O*-glycans may bind to MUC17 within the outer glycocalyx rather than engaging epithelial receptors required for stable adhesion or invasion. This contrasts with direct receptor-mediated adhesion, which is required for invasion. In experimental systems, increased surface expression of MUC17 significantly reduced bacterial attachment to epithelial cells, demonstrating that the net effect of these interactions is protective rather than permissive [7,73,77]. Furthermore, the apical membrane of epithelial cells can shed mucin-containing microvillus-derived vesicles, effectively removing bound bacteria from the epithelial surface and reinforcing this decoy function [77,78]. This mechanism differs in important ways from that of other transmembrane mucins, particularly MUC1. MUC1 has been shown to function as a receptor [79], where enteropathogens such as *Salmonella* bind to sialic acid presented by the mucin domain. Consequently, cell type-specific expression of glycosyltransferases, generating diverse glycoforms with distinct charge, branching, and terminal modifications, will shape the spectrum of microbial interactions with host cells by altering the repertoire of glycan ligands available. From an evolutionary perspective, variation within mucin TR regions and their glycosylation patterns likely reflects adaptation to microbial pressures, enabling fine-tuning of host-microbe interactions at the mucosal surface.

The glycocalyx architecture further imposes strict physical constraints on microbial access. The 30 nm mesh size effectively excludes bacteria, which typically exceed this dimension, preventing them from penetrating the glycocalyx and reaching the epithelial surface. This is supported by live imaging studies showing that bacteria are spatially segregated from the brush border in the presence of an intact MUC17-rich glycocalyx, whereas the genetic disruption of this barrier leads to increased bacterial contact with enterocytes [7,77].

## Formation of the MUC17-rich enterocyte glycocalyx during development

The enterocyte glycocalyx is not fully established at birth but undergoes a defined postnatal maturation, coinciding with major changes in diet, microbiota composition, and both innate and adaptive immune systems. A critical window for this process is the suckling-weaning transition in mammals, during which the intestinal epithelium adapts from a relatively protected, milk-fed environment to one exposed to complex dietary components and an expanding microbial ecosystem [80]. During this period, the emergence of a dense MUC17-rich glycocalyx marks a key step in the functional maturation of the epithelial barrier. This cell-attached barrier is especially important in the small intestine, where the mucus layer is loose and permeable to bacteria, in contrast with the colon, where the inner mucus layer forms a dense barrier that normally separates bacteria from the epithelium.

In the neonatal small intestine, the apical surface of enterocytes is devoid of a glycocalyx, with little or no MUC17 present at the brush border. As development progresses toward weaning, MUC17 expression is sharply up-regulated, both at the transcript and protein levels, leading to its accumulation at the distal tips of microvilli (Figure 2A) [7]. This dynamic increase in MUC17 coincides with the appearance of a structurally defined glycocalyx layer that extends into the intestinal lumen and physically separates luminal contents from the epithelial surface. The synchronization between MUC17 expression and glycocalyx formation strongly supports a causal role for MUC17 in establishing this barrier.

**Figure 2:**
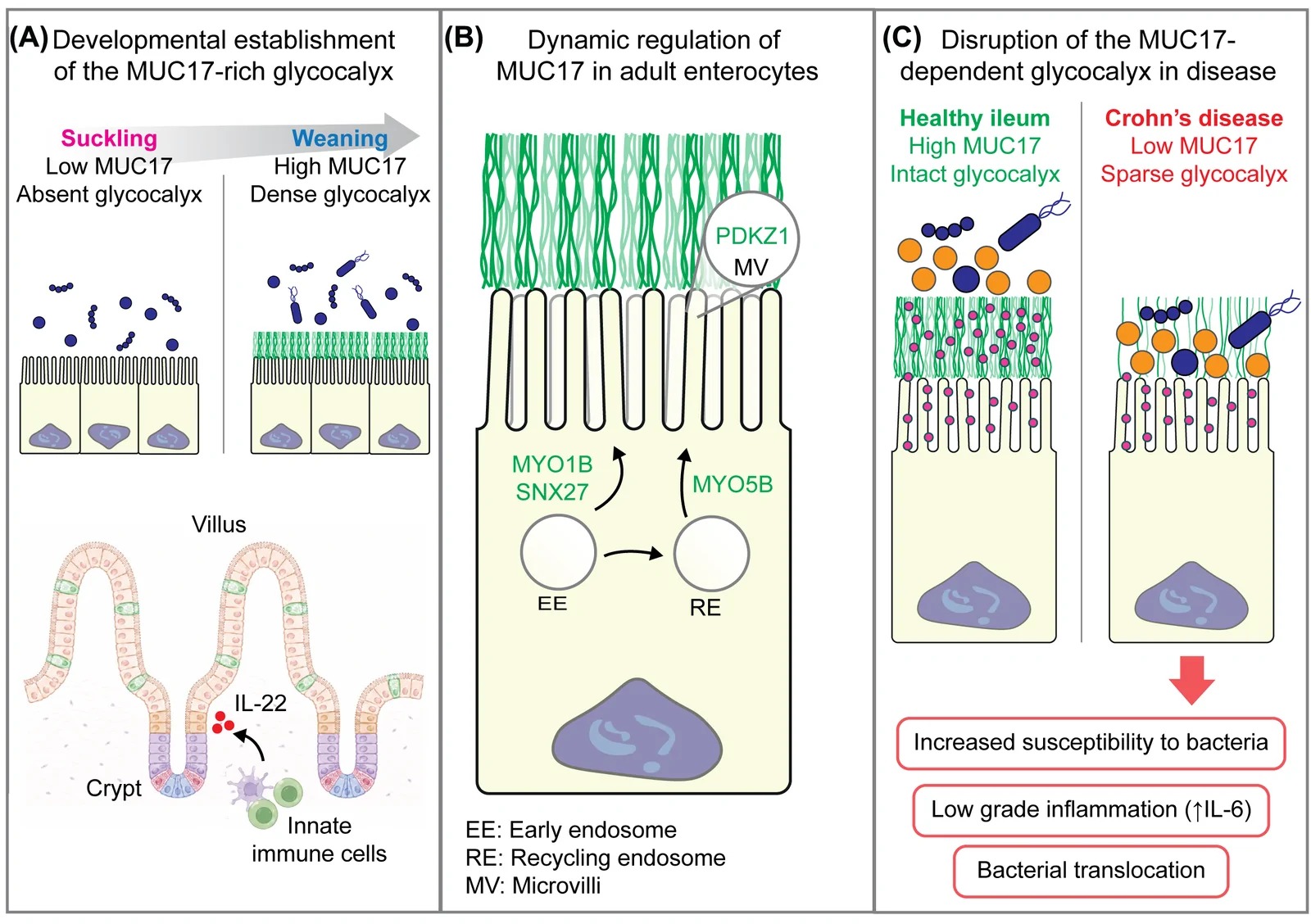
Regulation and disease relevance of the MUC17-rich enterocyte glycocalyx (**A**) Developmental establishment of the MUC17-rich glycocalyx during the suckling–weaning transition. IL-22 from innate immune cells promotes MUC17 expression and formation of a dense apical glycocalyx in enterocytes. (**B**) Dynamic regulation of MUC17 in adult enterocytes. MUC17 is retained at microvilli through PDZK1 and regulated by apical trafficking and recycling involving MYO1B, MYO5B, and SNX27. (**C**) Disruption of the MUC17-dependent glycocalyx barrier in Crohn’s disease results in a sparse glycocalyx, increased bacterial access to the epithelium, low-grade inflammation, and bacterial translocation across the epithelial barrier.

A key driver of this developmental program is the cytokine interleukin-22 (IL-22), which is produced by innate immune cells in response to microbial colonization and environmental cues during the suckling-weaning transition. IL-22 signaling induces MUC17 expression in enterocytes during the weaning period, promoting a state characterized by high MUC17 abundance at the apical membrane [7]. Notably, this induction is not uniform across the crypt-villus axis of the small intestinal epithelium but is associated with specific populations of postmitotic progenitor cells that are undergoing maturation into fully differentiated enterocytes (Figure 2A). This suggests that MUC17 expression is tightly integrated into endogenous epithelial differentiation programs as well as controlled by immune-derived signals in response to microbial changes in the small intestine.

Collectively, these findings position MUC17 as a central component of a developmentally programmed innate barrier system that is activated during early life. The coordinated up-regulation of MUC17, driven by IL-22 and aligned with epithelial differentiation, ensures that the glycocalyx is established precisely when the intestine becomes exposed to increased microbial and dietary challenges.

## Cellular trafficking, dynamics, and turnover of MUC17 within the enterocyte glycocalyx

The barrier function of MUC17 is inseparable from its precise localization and dynamic regulation at the apical brush border membrane of epithelial cells, particularly intestinal enterocytes. Despite its structural role, MUC17 within the glycocalyx is not static. Instead, the protein undergoes turnover, with renewal occurring on the order of 12–24 h, substantially faster than the lifespan of enterocytes, which is approximately 3–5 days [81]. This indicates that individual epithelial cells must replenish their glycocalyx throughout their life span in order to maintain barrier integrity in the face of constant exposure to mechanical stress, digestive enzymes, and microbial activity. As a consequence, MUC17 must be delivered and retained at the cell surface to maintain a dense and functional glycocalyx. This is achieved through tightly coordinated trafficking pathways coupled to turnover and recycling mechanisms.

The cytoplasmic tail of MUC17 plays a central role in regulating its localization and functional integration at the apical surface of enterocytes. A defining feature of this domain is a conserved class I PDZ-binding motif, which mediates interaction with the scaffolding protein PDZK1. This PDZ interaction is essential for maintaining MUC17 at the brush border membrane and anchors it within a broader apical protein network that coordinates membrane organization and stability (Figure 2B) [72]. Beyond anchoring, the MUC17 cytoplasmic tail is subject to post-translational regulation, including phosphorylation, which is thought to modulate its interactions with intracellular partners and trafficking machinery [73]. Although the precise phosphorylation sites and kinase(s) involved remain incompletely characterized, such modifications likely provide a mechanism for dynamically regulating MUC17 localization in response to cellular signals.

MUC17 trafficking is further controlled by a coordinated network of motor proteins and endosomal sorting factors (Figure 2B). Recent studies have identified the actin-based motors MYO1B and MYO5B, together with the PDZ domain-containing sorting nexin SNX27, as key regulators of MUC17 apical targeting and recycling [82]. MYO5B is implicated in the delivery of MUC17 to the apical membrane, while MYO1B contributes to maintaining its distribution and stability within the brush border. SNX27, in turn, facilitates the recycling of internalized MUC17 back to the plasma membrane, possibly linking endosomal trafficking to PDZ-mediated retention. Together, these components establish a trafficking axis that ensures sustained surface expression of MUC17 and, consequently, integrity of the glycocalyx. Importantly, the association between MUC17 and PDZK1 is not static but dynamically regulated in response to physiological stimuli. Upon stimulation of secretion, for example by cholinergic agonists, MUC17 dissociates from PDZK1 and is internalized from the apical membrane [83]. This process occurs in parallel with the internalization of the Na^+^/H^+^ exchanger NHE3 and the recruitment of the secretory CFTR to the membrane, reflecting coordinated remodeling of the apical surface.

The integration of trafficking and turnover mechanisms ensures that MUC17 contributes to a continuously renewed and spatially organized glycocalyx barrier. Disruption of these processes, whether through impaired apical targeting, defective recycling, or excessive internalization, leads to depletion of MUC17 at the cell surface and compromised glycocalyx density. In support of this hypothesis, experimental perturbation of MYO1B, MYO5B, or SNX27 results in altered MUC17 localization.

## The role of MUC17 in Crohn’s disease, inflammation, and epithelial repair

Crohn's disease is a chronic inflammatory bowel disease characterized by dysregulated host responses to the intestinal microbiota [84]. A substantial body of genetic and functional evidence implicates defective intracellular handling of bacteria in Crohn’s disease pathogenesis. For example, disease-associated variants in *NOD2*, which encodes an intracellular sensor of bacterial peptidoglycan, and *ATG16L1*, which encodes a key component of the autophagy machinery, link Crohn’s disease susceptibility to impaired bacterial recognition, xenophagy, and clearance of intracellular microbes [85,86]. In this context, Crohn’s disease-associated adherent-invasive *Escherichia coli* have attracted particular interest because these bacteria can adhere to and invade intestinal epithelial cells and survive intracellularly, including within macrophages [87,88]. These findings have led to the concept that Crohn’s disease involves abnormalities in the handling of intracellular bacteria.

MUC17 adds an additional, cell-surface dimension to this model. Rather than acting after bacteria have entered epithelial cells, MUC17 forms part of the apical glycocalyx that limits bacterial access to the enterocyte membrane in the first place. Disruption of MUC17 function has emerged as a defining feature of impaired small intestinal glycocalyx barrier with direct relevance to Crohn’s disease affecting the terminal ileum. In healthy ileum, MUC17 is positioned at the enterocyte brush border, where it forms a dense glycocalyx that separates luminal bacteria from the epithelial surface. In noninflamed ileal biopsies from patients with Crohn’s disease, MUC17 protein levels are significantly reduced at the brush border (Figure 2C) [77]. This selective spatial loss of MUC17 is accompanied by impaired glycocalyx barrier function, demonstrated by increased contact between luminal bacteria and the brush border. Thus, even in macroscopically noninflamed tissue, Crohn’s disease is associated with a molecular defect at the bacteria–epithelium interface. Notably, the absence of MUC17 from the brush border is not due to decreased MUC17 transcription [89], but rather an effect of impaired trafficking of MUC17 to the apical cell membrane. Thus, MUC17 dysfunction may represent an epithelial surface defect that precedes, and potentially increases the burden on, intracellular bacterial handling pathways such as NOD2- and ATG16L1-dependent responses.

This interpretation is supported by the Muc17ΔIEC mouse model, where Muc17 was absent from the tips of small intestinal microvilli and from microvillus-derived luminal vesicles, confirming effective loss of the transmembrane mucin [77]. Importantly, deletion of Muc17 did not cause compensatory changes in the abundance of other transmembrane mucins, such as Muc13, or the main intestinal gel-forming mucin, Muc2. Instead, bioorthogonal labeling of mucin-type *O*-glycans revealed a significant reduction in apical *O*-glycan density in the small intestinal brush border, suggesting that Muc17 deletion produces a specific loss of the small intestinal glycocalyx without broadly disrupting epithelial cell architecture.

Despite the absence of overt histological inflammation, Muc17ΔIEC mice displayed clear functional barrier defects. Under specific pathogen-free conditions, commensal bacteria were found in direct contact with the jejunal brush border significantly more often than in control wild-type littermates. The increased bacterial contact was accompanied by signs of altered epithelial homeostasis, including enhanced epithelial cell proliferation in the crypts and increased cell apoptosis at villus tips. Although classical histological inflammation was not apparent, IL-6 expression was significantly increased in the small intestine, indicating low-grade inflammatory activation likely driven by increased bacterial pressure at the epithelial surface. This subclinical barrier defect became more evident under infectious challenge. In particular, the Muc17ΔIEC phenotype was strongly exacerbated during infection with *Citrobacter rodentium*, a murine attaching-and-effacing pathogen that normally colonizes the distal colon. In Muc17-deficient mice, *C. rodentium* showed abnormally high small intestinal colonization, with approximately three orders of magnitude higher bacterial counts in the jejunal lumen and mucosa compared with wild-type controls at early infection [77]. These findings demonstrate that Muc17 is critical for restricting pathogen access to the small intestinal epithelium (Figure 2C). Importantly, the Muc17ΔIEC mouse represents a valuable experimental model for studying small intestinal infection in rodents, addressing a major limitation of existing models, which are largely restricted to investigating host responses in the distal intestine.

## Methodological aspects of capturing MUC17 function in the glycocalyx

Understanding the functional properties of the glycocalyx has required the development of experimental systems that preserve its native, hydrated structure. Conventional fixation approaches tend to collapse the hydrated mucin domain and obscure its function under truly physiological conditions, making live or minimally perturbed systems essential. A major advance in this context is the Glyco-TRAPP assay [90], which enables direct and quantitative assessment of glycocalyx permeability in intact tissues by leveraging whole-mount tissue imaging with fluorescent probes combined with time-lapse confocal microscopy. This method relies on tracking the diffusion of fluorescent molecules relative to the epithelial surface. By using dextrans of defined molecular size, it becomes possible to measure size-dependent exclusion, as large macromolecules are prevented from penetrating the glycocalyx while smaller molecules can diffuse more freely. The Glyco-TRAPP assay can be extended to include fluorescently labeled bacteria, allowing direct visualization of microbial behavior at the epithelial interface and quantification of bacterial contact with the glycocalyx and brush border.

## Therapeutic perspectives and future directions

The identification of MUC17 as a central determinant of glycocalyx integrity opens new avenues for therapeutic intervention in intestinal disease. In contrast with strategies that primarily target immune pathways, modulation of the epithelial barrier represents an upstream approach aimed at restoring the physical interface that separates host tissues from the luminal environment. This is particularly relevant in conditions such as inflammatory bowel disease, where increased epithelial permeability and excessive microbial contact are hallmarks of disease pathogenesis. Reinforcing the MUC17-dependent glycocalyx barrier could therefore reduce bacterial access to the epithelium, dampen inappropriate immune activation, and promote mucosal homeostasis. Similar principles may apply to enteric infections, where strengthening the glycocalyx barrier could limit pathogen adhesion and invasion. Several potential strategies can be envisioned to enhance or restore MUC17 function. One approach is to promote endogenous MUC17 expression through modulation of cytokine pathways, such as IL-22 signaling, which has been shown to induce MUC17 during epithelial maturation. Pharmacological activation of such pathways, or delivery of recombinant cytokines, could enhance glycocalyx formation in settings where it is compromised. Alternatively, targeting the intracellular trafficking machinery that governs MUC17 localization, such as PDZK1-mediated retention or SNX27-dependent recycling, may help restore proper apical presentation of MUC17 in diseased epithelia. In parallel, probiotic or microbiota-targeted therapies may indirectly support MUC17 function by shaping microbial communities that promote barrier integrity, for example through the production of metabolites that influence epithelial differentiation and glycosylation.

Despite these emerging opportunities, several key questions remain unresolved. The signaling functions of MUC17, particularly those mediated by its EGF-like domains and cytoplasmic tail, are incompletely understood and may reveal additional therapeutic targets. The extent to which MUC17 participates in bidirectional communication with the microbiota also remains to be defined, including whether specific microbial species or metabolites regulate its expression, glycosylation, or trafficking. Furthermore, while current knowledge is largely focused on the intestine, it is unclear whether MUC17 or related transmembrane mucins with corresponding glycocalyx mechanisms play analogous roles in other mucosal tissues, raising the possibility of broader systemic relevance. Finally, an important challenge lies in the development of robust biomarkers and functional assays to assess glycocalyx integrity in clinical settings. Recent methodological advances, such as live imaging-based permeability assays, provide a framework for quantifying glycocalyx function *ex vivo*, but their translation to routine clinical use will require further refinement. Such tools will be essential for patient stratification, monitoring therapeutic responses, and guiding the development of glycocalyx-targeted interventions.

In summary, MUC17 emerges as a central structural component of the intestinal glycocalyx, forming a dense, cell-attached barrier that defines the final interface between the epithelium and the luminal environment. Beyond its structural role, MUC17 operates as a dynamic and tightly regulated system, with its trafficking, turnover, and glycosylation enabling rapid adaptation to environmental and microbial cues. Thus, MUC17 is critical for maintaining spatial segregation between host tissues and the microbiota, thereby preserving epithelial integrity and preventing inappropriate immune activation. Its disruption is intricately linked to impaired barrier function, increased microbial contact, and intestinal disease, underscoring its importance in both homeostasis and pathogenesis. Collectively, MUC17 is positioned as a key determinant of mucosal defense, and the glycocalyx constitutes an essential, yet underappreciated, component of epithelial biology.

## Open Access

Open access for this article was enabled by the participation of the University of Gothenburg in an all-inclusive Read & Publish agreement with Portland Press and the Biochemical Society under an agreement with BIBSAM.

## Abbreviations

CF: cystic fibrosis
EGFR: EGF receptor
HER2: human epidermal growth factor receptor 2
IL-22: interleukin-22
PTS: proline, threonine, and serine
SEA: sea urchin sperm protein, enterokinase, and agrin
TRs: tandem repeats
